# Prevalence and Anthropometric Risk of Metabolic Syndrome in Taiwanese Adolescents

**DOI:** 10.5402/2011/743640

**Published:** 2011-07-13

**Authors:** Nain-Feng Chu, Hsien-Chuan Chin, Shu-Chuan Wang

**Affiliations:** ^1^Department of Community Medicine, Shuang-Ho Hospital, Taipei Medical University, Taipei, Taiwan; ^2^School of Public Health, National Defense Medical Center, No. 161, Sec. 6, Min-Chuan East Road, Nei-Hu 114, Taipei, Taiwan

## Abstract

*Background*. To evaluate the prevalence and the importance of anthropometric indexes on metabolic syndrome (MetS) among young adolescents in Taiwan. *Methods*. We conducted a cross sectional survey to obtain a representative sampling among Taipei adolescents in 2003, totally enrolled of 1,562 adolescents (764 boys and 798 girls) from age 11 to 15. We used modified NCEP-ATP III criteria to diagnose metabolic syndrome in young adolescents including: blood pressure ≧90th percentile, fasting glucose ≧90th, TG ≧ 90th, HDL-C ≦ 10th, and BMI or WC ≧ 90th according to age and gender specific recommendations. *Results*. The overall prevalence of MetS was 4.8% for boys and 3.9% for girls. BMI and WC were significantly associated with MetS for both boys and girls, even after adjusting for age, cigarette smoking, alcohol drinking and pubertal status. However, after further adjusting for BMI or WC, WC for boys (OR = 1.14, 95% CI = 1.05–1.24) and BMI for girls (OR = 1.36, 95% CI = 1.13–1.64) were significantly associated with MetS. *Conclusions*. Adolescents with abnormal BMI or waist circumference had 10 to 20 times higher odds of MetS when compared to normal subjects. Obesity, either general or central adiposity, may play an important role in the development of MetS among adolescents.

## 1. Background

In the last 30 years, socioeconomic growth and changes in life style patterns have been associated with the increasing prevalence and the clustering of chronic diseases. The main causes of death in Taiwan have shifted from infectious diseases to chronic diseases such as malignancies, cardiovascular disease, diabetic mellitus, and hypertension [1]. Insulin resistance, dyslipidemia, and abdominal obesity became important independent and emergent cardiovascular risk factors in developing and developed countries.

Metabolic syndrome (MetS) is a cluster of multiple cardiometabolic risk factors, and its components include abdominal obesity, dyslipidemia, high blood pressure (BP), insulin resistance, (impaired glucose metabolism or diabetes), and proinflammatory and prothrombotic status [2]. This clustering of several cardiometabolic risk factors was first proposed in 1920 [3]. However, it was not until 1998 that the World Health Organization (WHO) named these clustering characteristics as “insulin resistance syndrome” and defined its components and criteria [4]. After that, different definitions and criteria were proposed, such as the third report from the National Cholesterol Education Program (NCEP), the European Group for the Study of Insulin Resistance (EGIR), American Association of Clinical Endocrinologists (AACE), and the International Diabetes Federation (IDF) in 2005 [2, 5–7]. 

The risk factors for metabolic syndrome include age, gender, race, lifestyle, obesity, and heredity [8–15]. Many studies have shown that metabolic syndrome is positively associated with the morbidity and mortality from cardiovascular diseases and Type 2 diabetes in adulthood [12, 16–20]. The prevalence of MetS constantly increases with increased age [10] and is becoming one of the most important public health issues in the world.

However, metabolic syndrome can be traced not only in adults but also in childhood and adolescents [10]. In the past, the prevalence of MetS in adults has been investigated around the world; [11, 21–24] but recently, many studies focus on children and adolescents and use different criteria to discuss the prevalence of MetS [10, 25–29]. More important, for obesity and metabolic syndrome should be prevented as early as possible.

The purpose of this study is to evaluate the prevalence of MetS among young adolescents in Taiwan. Furthermore, this manuscript also address the importance of anthropometric indices, such as body mass index (BMI) and/or waist circumference (WC) which may represent different adiposity, to identify the odds of MetS among young adolescents in Taiwan.

## 2. Materials and Methods

### 2.1. Study Design and Sampling

We conducted a cross-sectional survey among junior high-school students in Taipei during the year 2003 to obtain a representative distribution of demographic, lifestyle, and biochemical characteristics among young adolescents. The sampling method and results have been described elsewhere [30]. Briefly, after a multistage sampling of 85 junior high-schools, we randomly selected 1,797 students for our survey. We excluded 235 subjects who refused to participate or had missing or incomplete data, so the final analyses included 1,562 subjects (a response rate around 87%) between 11–15-year-old (764 boys and 798 girls). This human subjects research was approved by the Institutional Review Board of our institution.

### 2.2. Study Methods

All participants completed a structured questionnaire that included dietary patterns, lifestyle, such as cigarette smoking and alcohol use, and pubertal status (by asking about the development of penis/testes and pubic hair for boys and breast growth and pubic hair for girls). Anthropometric measurements included body height, body weight, BMI, waist circumference, hip circumference, systolic blood pressure (SBP), and diastolic blood pressure (DBP) and were assessed by trained research technicians. 

Body height was recorded to the nearest 0.1 cm while subjects were barefoot and wearing light indoor clothing. Body weight was recorded to the nearest 0.1 kg using a segmental body composition analyzer (Tanita Corp., Tokyo, Japan) [31]. Body mass index (BMI) was calculated as weight in kilograms divided by the square of height in meters. Waist circumference was measured to the nearest 0.1 cm at the level of the midpoint between the inferior margin of the last rib and the iliac crest. Hip circumference was measured 4 cm below the anterior superior process of the iliac spine to the nearest 0.1 cm [32].

Blood pressure was measured on the right arm after patients rested for ten minutes in a sitting position, using an appropriately sized cuff of a HEM-740C automatic digital blood pressure monitor (Omron Corp., Tokyo, Japan) [33].

After 10–12 hours of fasting overnight, a 10 mL venous blood specimen was collected using venous containers. Serum concentrations of TG and glucose were measured with randox reagent on a Hitachi 7150 autoanalyzer (Hitachi, Tokyo, Japan) [34–36]. HDL-C was measured enzymatically with Daiichi reagent on an Olympus AU600 autoanalyzer (Olympus, Tokyo, Japan) [35].

### 2.3. Definition of Metabolic Syndrome

Since there is no clear definition of metabolic syndrome for adolescents in Taiwan, we modified the NCEP-ATP III criteria for our population. The cut-off points for each variable used age- and gender-specific 90th percentile criteria, except that HDL-C used the 10th percentile criteria among this study population. The presence of three or more of the following components were considered metabolic syndrome in study subjects (1) blood pressure (SBP or DBP) ≧90th percentile, (2) fasting glucose ≧90th percentile, (3) TG ≧90th percentile, (4) HDL-C ≦ 10th percentile, and (5) BMI or waist circumference ≧90th percentile. For adolescents, BMI and waist circumference may represent different anthropometric characteristics, so we used either BMI or waist circumference for general or central obesity surrogate markers to represent obesity status.

### 2.4. Statistical Analysis

We used mean and standard deviation (SD) to describe the distribution of anthropometric measures, blood pressure and biochemical levels. Frequency (*n*) and percentage (%) were used to determine the prevalence of abnormal lifestyle and metabolic syndrome. For comparison between the sexes, we used independent *t*-test for continuous variables and used the chi-square (*χ* ^2^) test to assess the association between categorical variables. We used multivariate logistic regression analysis to evaluate the odds ratio of anthropometric variables, such as BMI and waist circumference, in relation to metabolic syndrome after adjusting for age, cigarette smoking, alcohol consumption, and pubertal status. A two-tailed *P* value less than 0.05 was considered statistically significant. All statistical analyses were conducted using the statistical package SAS 8.2 (SAS institute Inc., Cary, NC, USA).

## 3. Results

The distribution of anthropometric variables, blood pressure, biochemical levels, and lifestyles in both genders are shown in Table 1. In general, boys were taller, heavier, had higher BMI, waist, and hip circumferences. Boys also had higher SBP and glucose levels than girls (*P* < 0.05). However, girls had higher HDL-C levels than boys (*P* < 0.05).

We further examined the clustering of these metabolic components among the study population in Table 2. For boys, the most prevalent clustering components were high TG, low HDL-C, and abnormal anthropometric variables (about 29.7%). In addition to these, the most frequent clustering components for girls were low HDL-C, high BP, and abnormal anthropometric variables (about 22.6%).

Table 3 shows the prevalence of metabolic syndrome and its components in subjects with normal versus abnormal BMI and normal versus abnormal waist circumference by gender. The overall prevalence of MetS for boys was 4.8%; and for girls was 3.9%. In boys, the prevalence of metabolic syndrome for those with normal BMI (10th ≦ BMI < 90th percentile) was 2.1%, and 29.3% for relatively higher BMI (≧90th percentile). The odds ratio (OR) of MetS was 13.7 (95%  CI = 7.5–25.1) for subjects with higher BMIs when compared to the normal BMI subjects. Also, the prevalence of metabolic syndrome among those with normal and higher waist circumference was 1.6% and 33.3%, respectively, and the odds ratio was 20.9 (95%  CI = 10.8–40.5). 

In girls, the prevalence of metabolic syndrome for normal BMI was 1.1%, and 28.4% for higher BMI. The odds ratio of MetS was 26.0 (95%  CI = 11.3–60.2) for higher BMI when compared to the normal BMI subjects. The prevalence of metabolic syndrome for normal and higher waist circumference was 1.1% and 25.5%, respectively, and the odds ratio was 23.0 (95%  CI = 10.0–23.0).

Multivariate logistic regression models to assess the association between BMI and/or waist circumference in relation to metabolic syndrome are shown in Table 4. For boys, after adjusting for age, cigarette smoking, alcohol consumption, and pubertal status, every increasing unit of BMI and every additional cm of waist circumference was significantly associated with metabolic syndrome, with OR = 1.43 (95%  CI = 1.30–1.55) and OR = 1.16 (95%  CI = 1.12–1.20), respectively. For girls, the odds ratio that increases of 1 unit BMI and 1 cm waist circumference would increase metabolic syndrome was 1.50 (95%  CI = 1.35–1.67) and 1.19 (95%  CI = 1.14–1.24), respectively. After further adjusting for BMI or waist circumference, waist circumference (central obesity) for boys (OR = 1.14, 95%  CI = 1.05–1.24) and BMI (general obesity) for girls (OR = 1.36, 95%  CI = 1.13–1.64) were the most significant anthropometric variables associated with metabolic syndrome.

## 4. Discussion

Metabolic syndrome is positively associated with the development of cardiovascular diseases and Type 2 diabetes in adults. Furthermore, the risk of metabolic syndrome can be traced not only in adults but also in childhood and adolescence. In our study, the prevalence of MetS was 4.8% in boys and 3.9% in girls. The subjects with higher BMI or waist circumference had 10 to 20 times higher odds of MetS when compared to those with normal BMI or waist circumference. Waist circumference and BMI were the most significant anthropometric variables associated with metabolic syndrome for boys and girls, respectively.

The prevalence of MetS varies in different populations, at about 20–40% in Western countries and 10–20% among Asian adults [22]. For American adults, the prevalence of Mets was 23.9% during the period from 1988 to 1994 [24]. Furthermore, the prevalence of MetS among US adolescents was 4.2% to 6.4% from 1988 to 1992 and 1999 to 2000 [10]. However, the NHANES III findings suggested that the prevalence of MetS was 9.2% in 1988–1994 [25].

In Taiwan, the prevalence of MetS is around 12.9% in adults [23]. The components of metabolic syndrome were also different between the genders. Males had higher TG, lower HDL-C, higher BP, and abnormal anthropometrics, and females had lower HDL-C, higher BP and abnormal anthropometrics [37]. The results of components of MetS were similar to our findings; however, abnormal blood pressure in boys was not so prevalent in this age group, which may be due to the relative youth of this study population. Therefore, the distribution and the clustering of MetS components are different among different age and gender groups.

Obesity is positively associated with the development of MetS, and this trend is consistent even among young adolescents. When using BMI ≧ 95th percentile as obesity, the prevalence of MetS among younger adolescents was 32.1%, compared to 7.1% among those with BMI between 85 and 95th percentiles [10]. Druet et al. have shown that by using the modified NCEP-ATP III as criteria, the prevalence of MetS among overweight students (25 ≦ BMI < 30) was 27.7% and among obese students (BMI ≧ 30) was 46.5% [38]. These results were also similar to our finding that the prevalence of MetS among those with higher BMI was 29.3% and 28.4% for boys and girls, respectively (it was 2.1% in boys and 1.1% in girls with normal BMI). This indicated that the prevalence of MetS among overweight and obese young adolescents were significantly higher than normal subjects. These high-risk populations should pay attention to preventing MetS from developing in the future.

Body fat distribution may have different clinical meanings in adults and children. BMI is an indirect assessment of total body fatness; however, waist circumference may represent the amount of abdominal adiposity. Many studies have shown that abdominal (central) adiposity, rather than general obesity, is a stronger independent risk factor for the occurrence MetS, CVD, and Type 2 diabetes for adults [22, 25]. However, during childhood or adolescence, general and central obesity may differ due to dynamic growth status. According to our study, after adjusting for BMI or waist circumference, the association between anthropometric indices and metabolic syndrome differed for boys and girls. For boys, waist circumference was the most significant variable associated with metabolic syndrome. However, among girls, BMI had the greater impact in terms of metabolic syndrome. In addition, the Pearson's correlation coefficients of BMI and waist circumference in relation to body fat were 0.79 and 0.81, respectively, in boys; it was 0.95 and 0.81 in girls (data not shown). These findings suggest that waist circumference for boys and BMI for girls had the more significant association with body fatness and so with metabolic syndrome. Nevertheless, due to the limited nature of our cross-sectional, further research would be required to explain the possible mechanism.

Metabolic syndrome is associated with the development of Type 2 diabetes and cardiovascular diseases, which contribute to the morbidity and mortality from these chronic diseases. Metabolic syndrome has become one of the important issues for public health and clinical medicine for preventing, not only in adults but children for the prevention of chronic diseases' development. This study indicated that abnormal BMI or waist circumference adolescents had 10 to 20 times higher odds of MetS when compared to normal subjects. Obesity, either general or central adiposity, may play an important role in the development of MetS among children and young adolescents. Further study may be indicated for obese, both general and central obese, young adolescents to evaluate the association between obesity and MetS for better understanding the possible mechanisms; furthermore, the effectiveness of weight control program on the prevention of obesity and MetS among young adolescents are needed in the future.

## Figures and Tables

**Table 1:** The general characteristics of young adolescents in Taiwan.

Variables	Boys (*n* = 764)				Girls (*n* = 798)			
	Mean ± s.d.	10th	50th	90th	Mean ± s.d.	10th	50th	90th
Age (years)*	13.0 ± 0.8				13.1 ± 0.8			
Body height (cm)***	163.6 ± 8.1	152.5	164.0	173.5	157.7 ± 5.6	151.0	157.5	164.5
Body weight (kg)***	56.0 ± 13.0	41.0	54.0	73.0	49.8 ± 9.8	39.3	48.5	62.0
BMI (kg/m^2^)***	20.8 ± 3.9	16.6	20.0	26.1	20.0 ± 3.4	16.5	19.4	24.3
Waist circumference (cm)***	71.6 ± 10.2	61.0	69.0	86.0	67.5 ± 8.0	59.0	66.0	78.0
Hip circumference (cm)	89.1 ± 9.1	79.0	88.0	100.0	88.5 ± 7.6	80.0	88.0	98.0
Systolic BP (mmHg)***	118.0 ± 15.4	100.0	118.0	136.0	113.0 ± 13.5	97.0	113.0	129.0
Diastolic BP (mmHg)	68.3 ± 11.4	56.0	68.0	82.0	67.8 ± 10.8	55.0	68.0	80.0
Triglyceride (mg/dL)^@^	67.9 ± 33.2	37.0	59.0	111.0	68.4 ± 29.1	41.0	62.0	102.0
HDL-cholesterol (mg/dL)***	50.0 ± 11.4	36.0	49.0	66.0	52.8 ± 12.1	39.0	51.0	69.0
Fasting glucose (mg/dL)**	89.1 ± 7.9	79.0	89.0	98.0	87.8 ± 8.0	78.0	87.0	98.0
Cigarette smoking^1^								
No (%)	78.3^$^				85			
Yes (%)	5.0				4.5			
Alcohol consumption^2^								
No (%)	78.7				86			
Yes (%)	0.5				0.7			
Puberty development ^3 ∗∗∗^								
Yes (%)	85.1				90.2			

^$^The number of cigarette smoking, alcohol consumed, and puberty development not equal to 100% may be due to missing or incomplete data.

^1^Cigarette smoking: No: never smoking, Yes: current or previous smoking.

^2^Alcohol consumption: No: drinking less than once per week, Yes: drinking ≧1 per week.

^3^Puberty development: Yes: with any one of the three Tanner Stage 1 signs.

*P < 0.05, **P < 0.01, ***P < 0.001 when boys compared with girls.

^@^TG was tested after log transformation.

**Table 2:** Frequency and clustering of the metabolic syndrome (MetS) components among young adolescents in Taiwan.

	Boys		Girls		
	*n*	(%)	*n*	(%)	*P*-value
Frequency of MetS component^@^	(*n* = 764)		(*n* = 798)		

Abnormal anthropometric^1^	100	13.1	115	14.4	0.502
Abnormal BP^2^	131	17.2	138	17.3	0.988
Abnormal TG^3^	79	10.3	85	10.7	0.861
Abnormal HDL^4^	85	11.1	87	10.9	0.964
Abnormal Glu^5^	88	11.5	94	11.8	0.916

Clustering of MetS components^#^	(*n* = 37)		(*n* = 31)		

All five components	3	8.1	2	6.5	0.830
Four components	6	16.2	10	32.2	0.207
TG + HDL + BP + OB	4	10.8	5	16.1	0.777
Glu + other three^6^	2	5.4	5	16.1	0.295
Three components	28	75.7	19	61.3	0.309
TG + HDL + BP	2	6.5	2	5.4	0.747
TG + HDL + OB	11	29.7	1	3.2	0.011
TG + BP + OB	7	18.9	3	9.7	0.470
HDL + BP + OB	0	0	7	22.6	0.008
BP + Glu + OB	3	8.1	4	12.9	0.804
Glu + other two^6^	5	13.5	2	6.5	0.586

^@^The number and percentage of frequency among all study population which may be overlapping.

^1^Abnormal anthropometric is defined as BMI or waist circumference ≧ 90th percentile.

^2^Abnormal BP is defined as systolic or diastolic blood pressure ≧ 90th percentile.

^3^Abnormal TG is defined as triglyceride ≧90th percentile.

^4^Abnormal HDL is defined as HDL-cholesterol ≦10th percentile.

^5^Abnormal Glu is defined as fasting glucose ≧ 90 percentile.

^6^Glu + other three is defined as abnormal glucose plus any other three components of metabolic syndrome; Glu + other two is defined as abnormal glucose plus any other two components of the metabolic syndrome.

^#^The number and percentage of clustering is only among those subjects that have metabolic syndrome.

*P values when boys compared with girls.

**Table 3:** Prevalence of the components of metabolic syndrome (MetS) among young adolescents with different anthropometric status in Taiwan.

	Total	BMI		Waist Circumference	
		10–90th	≧90th	10–90th	≧90th
	*n* (%)	*n* (%)	*n* (%)	*n* (%)	*n* (%)
Boys	(*n* = 764)	(*n* = 610)	(*n* = 82)	(*n* = 626)	(*n* = 81)

Components of the MetS^1^					
0	453 (59.3)	394 (64.6)	0 (0.0)	411 (65.6)	0 (0.0)
1	188 (24.6)	154 (25.3)	24 (29.3)	150 (24.0)	24 (29.6)
2	86 (11.3)	49 (8.0)	34 (41.4)	55 (8.8)	30 (37.1)
3	28 (3.6)	13 (2.1)	15 (18.3)	9 (1.4)	19 (3.4)
4	6 (0.8)	0 (0.0)	6 (7.3)	1 (0.2)	5 (6.2)
5	3 (0.4)	0 (0.0)	3 (3.7)	0 (0.0)	3 (3.7)

Girls	(*n* = 798)	(*n* = 643)	(*n* = 81)	(*n* = 631)	(*n* = 94)

Components of the MetS^1^					
0	454 (56.9)	393 (61.2)	0 (0.0)	395 (62.6)	0 (0.0)
1	214 (26.8)	186 (29.0)	19 (23.5)	173 (27.4)	30 (31.9)
2	99 (12.4)	56 (8.7)	39 (48.1)	56 (8.9)	40 (42.6)
3	19 (2.4)	4 (0.6)	15 (18.5)	4 (0.6)	15 (16.0)
4	10 (1.2)	3 (0.5)	7 (8.7)	3 (0.5)	7 (7.4)
5	2 (0.3)	1 (0.1)	1 (1.2)	0 (0.0)	2 (2.1)

^1^Components of metabolic syndrome include BMI or waist circumference ≧90th percentile, systolic or diastolic blood pressure ≧90th percentile, triglyceride ≧ 90th percentile, HDL-cholesterol ≦ 10th percentile, and fasting glucose ≧90 the percentile.

**Table 4:** Multiple logistic regression analyses of anthropometric indices on the risk of metabolic syndrome.

	Model A^1^		Model B^2^	
	OR	95% CI	OR	95% CI
Boys (*n* = 764)				

BMI	1.43	1.30–1.55	1.04	0.85–1.28
Waist circumference	1.16	1.12–1.20	1.14	1.05–1.24

Girls (*n* = 798)				

BMI	1.50	1.35–1.67	1.36	1.13–1.64
Waist circumference	1.19	1.14–1.24	1.05	0.97–1.15

^1^Model A: adjusted for age, cigarette smoking, alcohol consumption, and stage of puberty.

^2^Model B: further adjusted for BMI or waist circumference.

**Table 5:** Age- and gender-specific percentile cut-off points among study variables.

Variables	Boys	Girls
	(*N* = 764)	(*N* = 798)
12 YO	*n* = 243	*n* = 229
BMI	24.8	23.6
Waist	84.0	75.0
Systolic BP	131.0	128.0
Diastolic BP	79.0	79.0
Triglyceride	117.0	106.0
HDL-Chol	38.0	39.0
Fasting glucose	100.0	99.0
13 YO	*n* = 258	*n* = 297
BMI	25.8	24.7
Waist	85.0	78.0
Systolic BP	136.0	129.0
Diastolic BP	81.0	80.0
Triglyceride	109.0	102.0
HDL-Chol	37.0	40.0
Fasting glucose	97.0	97.0
14 YO	*n* = 263	*n* = 272
BMI	26.9	24.6
Waist	89.0	78.0
Systolic BP	139.0	129.0
Diastolic BP	85.0	79.0
Triglyceride	110.0	97.0
HDL-Chol	35.0	38.0
Fasting glucose	97.0	97.0
